# Congenital Zika syndrome: geographical access to the health care network

**DOI:** 10.11606/s1518-8787.2025059006562

**Published:** 2025-06-11

**Authors:** Danielle Amaral de Freitas, Mayumi Duarte Wakimoto, Reinaldo Souza-Santos

**Affiliations:** I Fundação Oswaldo Cruz Escola Nacional de Saúde Pública Sérgio Arouca Rio de Janeiro RJ Brasil Fundação Oswaldo Cruz. Escola Nacional de Saúde Pública Sérgio Arouca. Rio de Janeiro, RJ, Brasil; II Universidade Federal do Rio de Janeiro Instituto de Atenção à Saúde São Francisco de Assis Rio de Janeiro RJ Brasil Universidade Federal do Rio de Janeiro. Instituto de Atenção à Saúde São Francisco de Assis. Rio de Janeiro, RJ, Brasil; III Instituto Nacional de Infectologia Evandro Chagas Serviço de Vigilância em Saúde Rio de Janeiro RJ Brasil Instituto Nacional de Infectologia Evandro Chagas. Serviço de Vigilância em Saúde. Rio de Janeiro, RJ, Brasil; IV Fundação Oswaldo Cruz Escola Nacional de Saúde Pública Sérgio Arouca Departamento de Endemias Samuel Pessoa Rio de Janeiro RJ Brasil Fundação Oswaldo Cruz. Escola Nacional de Saúde Pública Sérgio Arouca. Departamento de Endemias Samuel Pessoa. Rio de Janeiro, RJ, Brasil

**Keywords:** Epidemiology, Zika Virus Infection, Maternal and Child Health, Comprehensive Health Care, Universal Access to Health Services

## Abstract

**OBJECTIVES:**

To analyze the geographic access of children with congenital Zika syndrome (CZS), residing in the city of Rio de Janeiro between 2015 and 2017, to rehabilitation services.

**METHODS:**

Ecological study, based on spatial analysis. Commute maps were constructed using QGis 3.36.3 and the distances, time and cost between rehabilitation services and the homes of children with CZS, in the city of Rio de Janeiro, reported between 2015 and 2017 in the Registry System of Public Health Events.

**RESULTS:**

47 children were identified, 6 died. Among the 41 survivors, 17 were followed up in primary and secondary health care (PHC and SHC), 10 only in PHC, 7 only in SHC, and 7 had no follow-up record. CZS cases were more frequent in the north and west zones of the city. 143 trajectories followed by the 36 children between home and a rehabilitation service were identified; 147 commutes, followed 1 to 106 times; the majority destined for 3 units (n = 17 – 47.0%). The largest number of trajectories and commutes were among residents in the north and west zones of the city, poorer regions, with greater distances, cost, and time spent traveling between home and the health unit.

**CONCLUSIONS:**

The variation in trajectories, the number of recorded commutes, and the number of units included in the trajectories suggest the complexity of the children’s clinical condition, from basic care in PHC to procedures with greater technological density in specialized care.

## INTRODUCTION

Congenital Zika syndrome (CZS) is characterized by malformations in various systems, mainly in the central nervous system, osteoarticular and ocular systems in children exposed to the Zika virus during pregnancy^1^, requiring comprehensive, specialized care and social support^2-4^

Complications have a variety of impacts: 1) on children and families, who need intensive care, who face increased expenses, especially in health care, and loss of income; 2) on the health care network (HCN), which faces increased demand for specialized services; and 3) on the economy, due to increased absenteeism, especially among mothers^2-4^

In Brazil, the epicenter of the CZS epidemic, 3,563 cases were confirmed between 2015 and 2020. Given the seriousness of the situation, it was necessary to structure a HCN that offers comprehensive care for children, from prenatal care to follow-up, with clinical, laboratory, and imaging diagnosis. However, there are barriers to access, such as fragmented care, a lack of communication between services, the absence of a referral and counter-referral network, and geographical access difficulties, due to the lack of adapted transportation and the cost of travel^2-7^

The Pan American Health Organization (PAHO) has warned of the risk of another arbovirus disease caused by the oropouche virus (OROV), which can also cause congenital malformations, making it essential to analyze access and accessibility to HCN for a rapid response to a possible public health emergency^8^

Rio de Janeiro, one of Brazil’s most populous cities, faces challenges of poor urban transportation that make it difficult for children with CZS to travel to rehabilitation units. This study aims to analyze the geographical access of these children, diagnosed between 2015 and 2017, to rehabilitation services in the municipality.

## METHODS

This is a spatial ecological study whose unit of analysis is the planning area (PA) of residence of children with CZS in the municipality of Rio de Janeiro (RJ), notified between 2015 and 2017 in the Public Health Events Registry System (RESP).

The municipality of Rio de Janeiro is geographically and demographically diverse, with a population of 6,211,223, a high population density and 23.0% of the population living in favelas. It has 239 primary health care units (PHC - 79.0% coverage), 31 emergency care units, 24 hospitals located mainly in the south and central zones. The municipality is made up of 160 neighborhoods, divided into five PHC: AP1 (downtown and port area) commercial region, made up of 5.0% of Rio’s population; AP2 (south zone) represents 14.0% of the population, luxury buildings and irregular occupation by low-income population coexist; AP3 (north zone) has 34.0% of the population and is home to one in two of the city’s favela residents (49.9% of the city); AP4 (west zone) has 18.0% of the population; AP5 (west zone) is a region furthest from the city center, home to 30.0% of the population and has a smaller hospital network.

### Notification of Congenital Zika Syndrome

Data on children with a confirmed diagnosis of CZS was collected from the RESP system^9^ and a description was made of the geographical distribution of the place of residence by PA and the level of care for children with CZS.

In order to analyze the care pathways^10,11^ of these children, the addresses their mothers’ homes and rehabilitation units in Rio de Janeiro were obtained. It is expected that they will have a link with PHC in the territory and that they will be referred for specialized consultations and exams, through the Vacancy Regulation System (SISREG), to health units closest to their homes, when available, or to reference units according to complexity^9^

The care pathway was traced based on care data extracted from the management spreadsheets of the Municipal Health Department (SMS) (childcare appointments, specialized care, rehabilitation or exams), and supplemented with information obtained from SISREG. Health establishment registration numbers (CNES) were included to obtain the addresses.

A spreadsheet was drawn up with the geographical coordinates of the centroids of the neighbourhoods of residence and the addresses of the health units traveled to the specialized unit for rehabilitation or examination, obtained using Google Maps. Each journey between the neighborhood of residence and the specialized unit was considered a geographic path, which could have one to three health units, considering the intermediate units until reaching the final rehabilitation unit. Each patient may have different journeys, traveled at different times, and the same journey may have been traveled more than once. Between the beginning (neighborhood of residence) and the end (unit that performed the procedure), the child may have passed only through the PHC unit of reference, only through a PHC unit that requested the procedure in SISREG or both. The number of times each journey was traveled and how many units were visited on the journeys was calculated.

To construct the commutes, the QGis 3.36.3 program’s command for transforming points into lines was used. During the generation of the line maps, a variable with the number of times each path was traveled was incorporated into each data sheet.

Commute maps were generated between the neighborhoods where children with CZS live and the health units they go to for rehabilitation, specialist consultations and tests. The units of analysis used were neighborhoods. The centroids of the neighborhoods of residence were used on the map to mitigate the risk of identifying addresses. In this way, each journey includes all the units traveled and the commutes represent the movement between the residence and the rehabilitation unit. To better visualize the results, a map was generated for each PA of residence.

Using the Google Maps application and the geographic coordinates of the children’s addresses, the time, distance and cost to travel from the home to the rehabilitation unit were calculated. To complement the other results, the metrics were aggregated by PA of residence. The calculation was carried out on business days, at 8am, since the travel time provided by the app takes traffic into account. Time and cost were calculated based on the use of public transport and, for distance, on travel by car. The cost was stratified according to the value of the public transport ticket in dollars, with a value of 81 cents.

The digital grid of census tracts was obtained from the website of the Brazilian Institute of Geography and Statistics (IBGE)^12^ to make the digital grid of neighborhoods and PAs.

The project was approved by the Ethics Committees of the Escola Nacional de Saúde Pública Sergio Arouca/Fundação Oswaldo Cruz (CAAE: 94162218.5.0000.5240) and the Rio de Janeiro SMS (CAAE: 94162218.5.3001.5279).

## RESULTS

**Figure 1:**
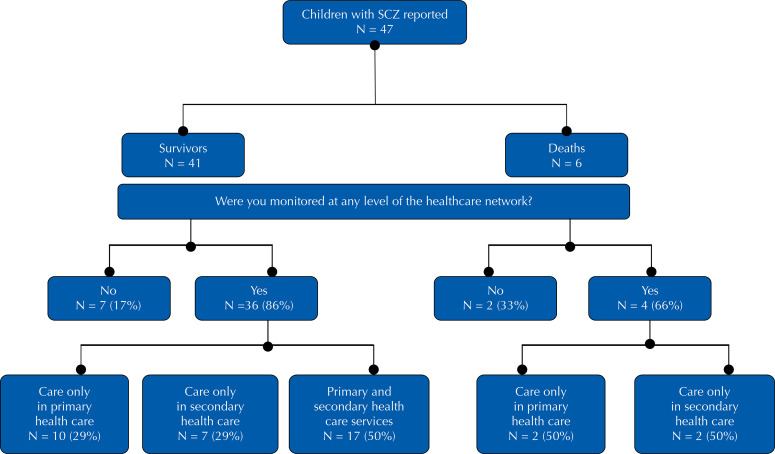
Attendance of children with congenital Zika syndrome according to level of care, residents of the municipality of Rio de Janeiro, 2017.

Source: Public Health Event Registration System and Rio de Janeiro Municipal Vacancy Regulation System.

PHC: primary health care; SHC: secondary health care.

The municipality of Rio de Janeiro had 47 children with CZS, most of them living in the north (AP3) and west (AP5) zones; of which 13.0% (n= 6) died. Among the 41 children who survived, 83.0% (n= 34) had records of childcare (n= 27, 79.0%) and/or specialist follow-up (n= 24, 71.0%); and 17.0% (n= 7) had no record of care at any of the health care levels. Only 50% (n= 17) had a record of follow-up at both levels of health care, 29.0% (n= 10) only in PHC and 21.0% (n= 7) only in specialized care (Figure 1).

The number of journeys made by each of the 36 children varied between one and 19. 67.0% (n= 24) made between one and four journeys, while 33.0% (n= 11) of the children made five or more journeys (Table 1). The median was two journeys, and the average was four journeys between home and a rehabilitation service for all the children. A total of 143 journeys were identified by the children with CZS, of which each had one to three specialized units as their destination: 47.0% (n= 17) to three units, 28.0% (n= 10) to two units and 25.0% (n= 9) to just one unit. As for the journeys: 61.0% (n= 87) to three units, 31.0% (n= 45) to two units, and 8.0% (n= 11) to one unit (Table 2).

**Table 1:** Number of destination units and journeys traveled by children with congenital Zika syndrome living in the municipality of Rio de Janeiro, 2015 to 2018.

	1 unit		2 units		3 units		Children	Total
	n	%	n	%	N	%	n	%
Children/units	9	25	10	28	17	47	36	100
Journeys								
1 a 4	9		13		15		24	67
5 a 8	1		9		19		5	14
9 a 12	1		22		23		6	17
13 a 19	0		1		18		1	2
Total	11	8	45	31	87	61	36	100

Source: Public Health Event Registration System and Rio de Janeiro Municipal Vacancy Regulation System.

**Table 2:** Number of commutes traveled by children with congenital Zika syndrome living in the municipality of Rio de Janeiro, by planning area, 2015 to 2018.

Program areas	Commutes								
	1		2 to 5		6 to 50		51 to 106		Total
	n	%	n	%	n	%	n	%	N
1	13	81.25	3	18.75	0	0	0	0	16
2	1	100	0	0	0	0	0	0	1
3	43	69.35	19	30.65	0	0	0	0	62
4	8	80.00	2	20.00	0	0	0	0	10
5	31	53.45	20	34.48	6	10.34	1	1.72	58
Total	96	65.00	44	30.00	6	4.00	1	1	147

Source: Public Health Event Registration System and Rio de Janeiro Municipal Vacancy Regulation System.

Of the 143 journeys, 147 commutes were recorded: 65.0% (96) were traveled only once; 30.0% (44) between two and five times; 4.0% (six) between six and 50 times; and 1.0% (one) between 51 and 106 times. The distribution of commutes according to area of residence was heterogeneous, with a predominance of AP3 and AP5 (Table 2).

**Figure 2:**
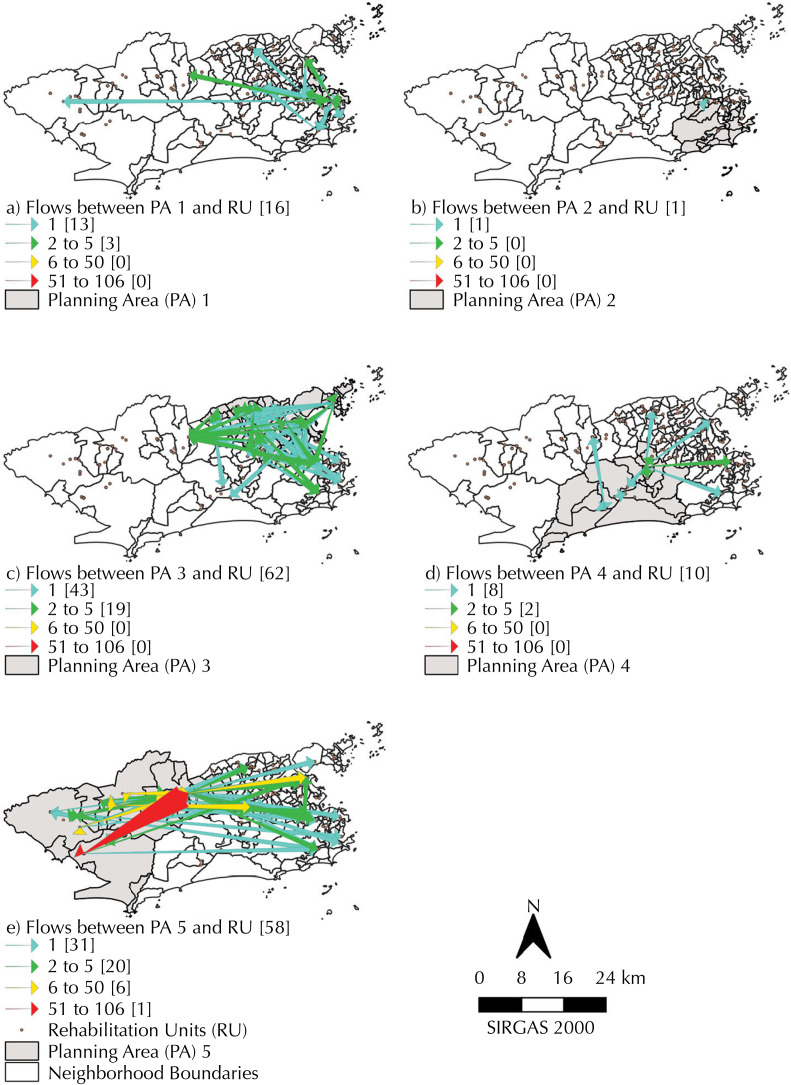
Map of the commute of children with congenital Zika syndrome between areas of residence planning and care rehabilitation units in the municipality of Rio de Janeiro, between 2015 and 2018.

PA: planning areas; UR: rehabilitation units.

Among the children living in AP1, 16 commutes were observed, of which 13 commutes were traveled only once, and three commutes were traveled between two and five times. The specialized units with the highest number of commutes were in districts far from AP1, in the south and north and only one, with two to five commutes in the west (Table 3, Figure 2a).

**Table 3:** Time spent, distance traveled, and cost of journeys made by children with congenital Zika syndrome living in the municipality of Rio de Janeiro, according to planning area, 2015 to 2018.

	Planning areas				
	AP1	AP3	AP4	AP5	Total
	**n (%)**	**n (%)**	**n (%)**	**n (%)**	**n (%)**
Time					
Up to 30 minutes	0	4 (7)	0	3 (5)	7 (5)
31 to 60 min	5 (42)	17 (30)	2 (25)	4 (7)	28 (21)
61 to 90 min	6 (50)	26 (46)	3 (25)	14 (25)	48 (36)
91 min or more	1 (8)	10 (6)	6 (75)	35 (63)	50 (38)
Distance					
Up to 10 km	4 (33)	9 (16)	3 (38)	5 (9)	21 (16)
11 to 20 km	5 (42)	26 (46)	0	2 (4)	33 (25)
21 to 30 km	1 (8)	13 (23)	5 (63)	11 (20)	30 (23)
31 to 40 km	1 (8)	9 (16)	0	6 (11)	16 (12)
41 to 50 km	0	0	0	10 (18)	10 (8)
50 km or more	1 (8)	0	0	22 (39)	23 (17)
Cost					
1 ticket	2 (17)	12 (21)	2 (25)	3 (5)	19 (14)
2 tickets	0	20 (35)	1 (13)	11 (20)	32 (24)
3 tickets	2 (17)	18 (32)	3 (38)	14 (25)	37 (28)
4 tickets	4 (33)	7 (12)	1 (13)	16 (29)	28 (21)
5 tickets or more	4 (33)	0	1 (13)	12 (21)	17 (13)

Source: Public Health Event Registration System and Rio de Janeiro Municipal Vacancy Regulation System.

In AP2, there was only one commute recorded, which was done once, to a neighborhood near the child’s place of residence (Figure 2b).

Children living in AP3 had 62 different commutes, 43 of which were traveled only once and 19 between two and five times. Most of the commutes were between neighborhoods that make up the PA itself, however the commutes traveled the most times were towards the south and west of the city (Table 3, Figure 2c).

Among the residents of AP4, 10 commutes were observed, eight of which were traveled only once and two of which were traveled between two and five times. Most of the commutes took place in the AP4 neighborhoods, but one journey took place more often in the central zone (Table 3, Figure 2d).

Among the children living in AP5, the highest number of commutes was recorded among the PHC in Rio de Janeiro, with 58 different journeys, 31 of which were traveled only once, 20 commutes traveled between two and five times and a single commute between 51 and 106 times. A single child has taken the same journey 106 times. The trajectories were widely dispersed, heading towards specialized units in the districts that make up the PA and towards the north, south and central zones (Table 3, Figure 2e).

The median time, distance and cost of the journeys between the home addresses of the children with CZS were 1 hour and 18 minutes, 22.6 kilometers and US$ 2.44, respectively (corresponding to up to three bus tickets).

In terms of time, 74.0% (n= 98) of the journeys lasted more than 1 hour (61 min), of which 50.0% (49) were among children living in Rio de Janeiro’s AP5, followed by residents of the northern zone with 37.0% (n= 36) of the journeys (Table 3).

The majority (64.0%) of journeys were up to 30 km. The same profile occurred among residents of AP1, 3 and 4. However, the longest distances (over 40 km) were covered by residents of AP5, with 24.0% of the journeys (n= 32) (Table 3).

In terms of cost, 62.0% (n= 82) of the journeys cost families more than two tickets. The families with the most expensive journeys were those living in AP5, where 50.0% (n= 28) of the journeys in the region cost more than US$12 per journey, representing 21.0% of all journeys in this price range (Table 3).

## DISCUSSION

Our study revealed inequities in access to health care, especially specialized care, aggravated by the great needs of children with CZS. This difficulty is more pronounced for those living in the poorer northern and western areas of Rio de Janeiro. In these areas, travel in search of care is more frequent, the distances between homes and health units are longer and both the cost and the time spent by children and their families are greater, impacting on access to the necessary care

Children under the age of one should be guaranteed childcare up to seven days after birth at the PHC^9^ . However, 34.0% (14) of children with CZS had no record of follow-up at the PHC. Children with CZS depend on procedures that vary from lower technological density to specialized care^9,13^ . In the context of the HCN, secondary and tertiary level units are responsible for planning and carrying out medium and high complexity actions, with specialized professionals and technological resources for diagnostic and therapeutic support, which are not available in PHC^14^

One study found insufficient coordination of care, longitudinality, comprehensiveness, and family orientation in PHC for children with CZS^15^. In our study, we found seven children with no record of care, either in PHC or in specialized services, indicating inadequate management and follow-up by the local manager^16,17^.

Only 17 children were followed up by PHC and the specialized network, as recommended by the World Health Organization (WHO)^9,13^. There is evidence that follow-up and early stimulation reduce sequelae, increase independence in basic functions and improve quality of life^18^ . In the state of Rio de Janeiro, 54.3% of them were being followed up by PHC and 60.3% in some specialized care^19^. In two cities in northeastern Brazil, weaknesses were found in the follow-up of pregnant women due to insufficient coverage of prenatal care and lack of coverage of health services with specialized care^20^. It is suggested that there is inadequate care management and regionalization process. The regionalization process presupposes the planning of strategic actions at regional level, as well as reducing inequities not only in terms of the spatial distribution of health units, but also in terms of regulating more equal access for users, in accordance with effective public policies^21^.

With the appearance of CZS, the flow of care in the municipality of Rio de Janeiro was organized, including Rehabilitation Centres (CER) and units offering various health services. However, even though the CER offers a wide range of services, the state of Rio de Janeiro does not have a CER with all types of rehabilitation^22^. As a result, children with alterations in various systems, such as those with CZS, need to go to various units for comprehensive care.

A study analyzing the spatial distribution of rehabilitation units in the state of Minas Gerais found a disproportion between rehabilitation modalities and inequitable distribution, reflecting care gaps^23^. It is therefore necessary to implement strategies to reduce the mobility of children with CZS in search of care by: assessing the availability of rehabilitation units, the distribution of units and services offered and increasing vacancies in health regions according to the proportion of people with special needs in the territory.

Our study found 143 different journeys taken by children with CZS, according to SMS and SISREG records, which may be underestimated. The children made between one and 19 journeys, but the majority made between one and four journeys, which means that the majority made fewer journeys, but those who made more journeys may have greater clinical involvement. Only one of them completed 19 journeys, perhaps because she was the most severe and had made more visits to the health unit.

Most of the children visited three health units, which may be related to the need for services and professionals from different specialties, not always available in the same health unit, which was also observed in the study by Duttine et al.^5^. This situation increases the need to travel in search of care, which can be an unfavorable factor for access to care.

Although there were many journeys, 65.0% were made only once and 30.0% up to five times, showing that various health services were part of the referral logistics. Our results correspond to the record of formal referral networks, but informal support networks are pointed out, which can increase access, although it can fragment and weaken the HCN, SISREG, with risk classification and equal access^5^

As for the distribution of CZS cases, AP3 and AP5 had the highest number of confirmed cases. These are poor areas, with unequal income distribution and high social vulnerability^24^. A study that assessed the spatial risk of developing CZS found that AP3 has a high relative spatial risk, reaching up to 7.86^24^ . Other studies have found an association between a higher prevalence of CZS and more vulnerable regions with inadequate access to basic sanitation and social disparities^6,25,26^

Children living in all the program areas were more likely to travel within their own area of residence, probably because it facilitates access. Although this is an important factor for the regionalization of care, since people are travelling shorter distances to be seen, this scenario was not observed in AP5^27^, in the west of the city, which is further away (in km) from the city center and has limited availability of health services. Therefore, among the children living in AP5 there were more trips outside the area and a greater number of commutes.

Even though most of the journeys were within the same area of residence, 74% of the children took more than 1 hour between home and the place of treatment, especially those living in AP3 and AP5, areas with the highest number of cases and the lowest social development indexes in the city^24^

Most journeys cost two tickets, totaling four tickets to and from health facilities. This means that, at the updated price for July 2024, on average, if the child traveled three times a week, the cost would be 5.0% of the minimum wage (US$ 266.62). A high figure, considering the socio-economic profile of families of children with CZS^3,6,28^.

Families face an exhausting routine of caring for children who have alterations such as difficulty swallowing, sleeping and resting, and developmental delays, causing caregiver overload, which can be aggravated by the fragmentation of care in the health network^3^. Childcare is generally provided by mothers who dedicate themselves entirely, with no income and sometimes abandoned by the children’s fathers, most of whom are from a less privileged class, black and brown, and live in precarious places with lower levels of social development^24,29^

Measures to mitigate the inadequate access to the HCN and the intense displacement of children with CZS should be implemented, such as: 1) establishing a commute of care that integrates all points of the HCN; 2) matrix support by family health teams, for comprehensive care linked to other levels of health care; and 3) investment in technologies for interconsultations, teleconsultations, guided consultations and telehealth.

As this was an ecological study, it was not possible to assess individual issues such as the clinical situation of the children, the socio-economic level of the families and the use of other health services without registration by the local health authorities. Another limitation refers to the estimation of transportation costs due to the lack of individual data. Considering the complexity of the logistics involved in transporting these children, families may spend even more on private rather than public transportation. Although other forms of non-formal referral may have occurred, SISREG should organize demand in an equitable, transparent and safe way, with risk classification, so that children have equal access to the network according to their health needs^30^, so it is the formal reference for regulation. Finally, considering the study’s approach, the use of secondary data limits control over the collection and quality of information.

## CONCLUSIONS

The variation in pathways, the number of commutes recorded, and the number of units included in each child’s journey suggest the complexity of the children’s clinical condition, from basic care in PHC to specialized care procedures. This movement also suggests a network that is still fragmented and requires organized care commutes, integrated and shared between all points of the HCN.

The evidence points to the possible severity of cases of vertical transmission of OROV, with neurological impairment, as observed in children with CZS, which highlights the need to structure the HCN to accommodate children with congenital syndromes.

Managers together with society should establish plans in social control bodies to develop strategies to better meet the demands of children with CZS, avoiding long journeys, subsidizing public transport, creating care units closer to homes, establishing the role of PHC with family health teams and multiprofessional teams in PHC (eMulti)^31^, to establish singular and targeted care, with shared linkage and coordination of care.

The social support network also needs to be interwoven into the commute of care, guaranteeing these families their constitutional right, either by establishing a social support network or through an effective cash transfer system, to help improve their quality of life.

Given the indicative nature of our results, individual observational studies are needed to elucidate the causality for these figures, which are a snapshot of what happens in other regions of the country.
