# Supplementary material for: Congenital Zika syndrome: geographical access to the health care network
Source: Rev Saude Publica. 2025 Jun 11;59:e10. doi: 10.11606/s1518-8787.2025059006562 (PMC12211770; doi:10.11606/s1518-8787.2025059006562)
Supplement: Translation

# Translation: Síndrome congênita da zika: acesso geográfico à rede de atenção à saúde

**Authors:** Danielle Amaral de Freitas, Mayumi Duarte Wakimoto, Reinaldo Souza-Santos

**Affiliations:** I Brasil Fundação Oswaldo Cruz. Escola Nacional de Saúde Pública Sérgio Arouca. Rio de Janeiro, RJ, Brasil; II Brasil Universidade Federal do Rio de Janeiro. Instituto de Atenção à Saúde São Francisco de Assis. Rio de Janeiro, RJ, Brasil; III Brasil Instituto Nacional de Infectologia Evandro Chagas. Serviço de Vigilância em Saúde. Rio de Janeiro, RJ, Brasil; IV Brasil Fundação Oswaldo Cruz. Escola Nacional de Saúde Pública Sérgio Arouca. Departamento de Endemias Samuel Pessoa. Rio de Janeiro, RJ, Brasil

**Keywords:** Epidemiologia, Infecção por Zika Vírus, Saúde Materno-Infantil, Assistência Integral à Saúde, Acesso Universal aos Serviços de Saúde

## Abstract

**OBJETIVO:**

Analisar o acesso geográfico das crianças com a síndrome congênita da zika (SCZ), diagnosticadas entre 2015 e 2017 residentes do município do Rio de Janeiro, aos serviços de reabilitação.

**MÉTODOS:**

Estudo ecológico, pautado na análise espacial. Foram construídos mapas de fluxo utilizando o QGis 3.36.3 e calculadas as distâncias, o tempo e o custo entre os serviços de reabilitação e as residências das crianças com SCZ, no município do Rio de Janeiro, notificadas entre 2015 e 2017 no Sistema do Registro de Eventos em Saúde Pública.

**RESULTADOS:**

Foram identificadas 47 crianças, seis evoluíram ao óbito. Entre as 41 sobreviventes, 17 foram acompanhadas na atenção primária e na atenção secundária à saúde (APS e ASS), 10 somente na APS, sete apenas na ASS e sete não tinham registro de acompanhamento. Os casos de SCZ foram mais frequentes nas zonas norte e oeste da cidade. Foram identificados 143 trajetos percorridos pelas 36 crianças entre a residência e algum serviço de reabilitação; 147 fluxos, percorridos de uma a 106 vezes; a maioria com destino a três unidades (n = 17 – 47,0%). A maior quantidade de trajetos e fluxos foram entre residentes nas zonas norte e oeste da cidade, regiões mais pobres, com maiores distâncias, custo e tempo gasto no percurso entre a residência e a unidade de saúde.

**CONCLUSÕES:**

A variação de percursos, a quantidade de fluxos registrados e o número de unidades incluídas nas trajetórias sugerem a complexidade da condição clínica das crianças, desde cuidados básicos na APS a procedimentos com maior densidade tecnológica na atenção especializada.

## INTRODUÇÃO

A síndrome congênita da zika (SCZ) é caracterizada por malformações em diversos sistemas, principalmente no sistema nervoso central, osteoarticular e ocular em crianças expostas ao vírus Zika durante a gestação^1^, demandando cuidados integrais, especializados e apoio social^2^.

As complicações geram variados impactos: 1) para as crianças e famílias, que precisam de cuidados intensivos, que enfrentam aumento de despesas, principalmente com saúde, e perda de rendimentos; 2) para a rede de atenção à saúde (RAS), que enfrenta aumento na procura por serviços especializados; e 3) para a economia, pelo aumento do absenteísmo, sobretudo das mães^2^.

No Brasil, epicentro da epidemia de SCZ, entre 2015 e 2020 foram confirmados 3.563 casos. Diante da gravidade, foi necessária a estruturação de uma RAS para oferecer cuidado integral às crianças, desde o acompanhamento pré-natal até o seguimento, com diagnóstico clínico, laboratorial e por imagem. Entretanto, há barreiras de acesso, como assistência fragmentada, falta de comunicação entre os serviços, ausência de uma rede de referência e contrarreferência, e dificuldade de acesso geográfico, devido à falta de transporte adaptado e ao custo de deslocamento^2^.

A Organização Pan-Americana da Saúde (OPAS) alertou sobre o risco de outra arbovirose causada pelo vírus oropouche (OROV) em 2024, que também pode gerar malformações congênitas, tornando essencial a análise do acesso e da acessibilidade à RAS para uma resposta rápida a uma possível emergência de saúde pública^8^.

O Rio de Janeiro, uma das cidades mais populosas do Brasil, enfrenta desafios de transporte urbano deficitário que dificultam o deslocamento de crianças com SCZ para as unidades de reabilitação. Este trabalho objetiva analisar o acesso geográfico dessas crianças, diagnosticadas entre 2015 e 2017, aos serviços de reabilitação no município.

## MÉTODOS

Trata-se de um estudo ecológico espacial tendo como unidade de análise a área de planejamento (AP) de residência das crianças com SCZ, no município do Rio de Janeiro (RJ), notificadas entre 2015 e 2017 no Sistema do Registro de Eventos em Saúde Pública (RESP).

O município do Rio de Janeiro apresenta grande diversidade geográfica e demográfica, com população de 6.211.223, elevada densidade demográfica e 23,0% da população vivendo em favelas. Possui 239 unidades de atenção primária à saúde (APS – 79,0% cobertura), 31 unidades de pronto atendimento, 24 hospitais localizados principalmente nas zonas sul e centro. O município é constituído por 160 bairros, dividido em cinco AP: AP1 (centro e portuária) região comercial, formada por 5,0% da população carioca; AP2 (zona sul) representa 14,0% da população, convivem as edificações de luxo e a ocupação irregular por população de baixa renda; AP3 (zona norte) detém 34,0% da população, é onde está um em cada dois moradores de favela da cidade (49,9% da cidade); AP4 (zona oeste) possui 18,0% da população; AP5 (zona oeste) é uma região mais afastada do centro da cidade, que abriga 30,0% da população e possui menor rede hospitalar.

### Notificação de síndrome congênita da zika

Foram coletados os dados das crianças com diagnóstico confirmado para SCZ no sistema RESP^9^ e foi realizada a descrição da distribuição geográfica do local de residência por AP e o nível de atenção para o atendimento das crianças com SCZ.

Para análise dos percursos assistenciais^10,11^ dessas crianças, foram obtidos os endereços dos domicílios das mães e das unidades de reabilitação no Rio de Janeiro. Espera-se que elas tenham vínculo com a APS no território e que sejam encaminhadas para consultas especializadas e exames, por meio do Sistema de Regulação de Vagas (SISREG), para unidades de saúde mais próximas às residências, quando disponíveis, ou para unidades de referência de segundo a complexidade^9^.

O percurso assistencial foi traçado a partir dos dados assistenciais extraídos das planilhas gerenciais da Secretaria Municipal de Saúde (SMS) (consultas de puericultura, atendimento especializado, reabilitação ou exames), e complementados com as informações obtidas no SISREG. Foram incluídos os números de Cadastro dos Estabelecimentos de Saúde (CNES) para obtenção dos endereços.

Foi elaborada uma planilha com coordenadas geográficas dos centroides dos bairros de residência e endereços das unidades de saúde percorridas até a unidade especializada para reabilitação ou exame, obtidos com uso do *Google Maps*. Cada percurso entre o bairro de residência e a unidade especializada foi considerado um trajeto geográfico, podendo este possuir de uma a três unidades de saúde, considerando as unidades intermediárias até a chegada à unidade final de reabilitação. Cada paciente pode possuir diferentes trajetos, percorridos em momentos diferenciados, e um mesmo trajeto pode ter sido percorrido mais de uma vez. Entre o início (bairro de residência) e o final (unidade que executou o procedimento), a criança pode ter passado apenas pela unidade de APS de referência, apenas por uma unidade de APS que solicitou o procedimento no SISREG ou por ambas. Foi calculado o número de vezes que cada trajeto foi percorrido e quantas unidades foram visitadas nos trajetos.

Para a construção dos fluxos, foi utilizado o comando de transformação de pontos em linhas do programa QGis 3.36.3. Durante a geração dos mapas de linhas, foi incorporada a cada planilha de dados uma variável com o número de vezes que cada trajeto foi percorrido.

Foram gerados mapas de fluxo entre os bairros das residências das crianças com SCZ e as unidades de saúde de referência para reabilitação, consulta com especialista e locais de realização de exames. As unidades de análise utilizadas foram bairros. Os centroides dos bairros de residência foram utilizados no mapa para mitigar o risco de identificação dos endereços. Dessa forma, cada trajeto inclui todas as unidades percorridas e os fluxos representam o deslocamento entre a residência e a unidade de reabilitação. Para melhor visualização dos resultados, foi gerado um mapa para cada AP de residência.

Com o uso do aplicativo *Google Maps,* utilizando as coordenadas geográficas dos endereços das crianças, foram calculados o tempo, a distância e o custo para o deslocamento da residência até a unidade de reabilitação. Para análise complementar aos demais resultados, as métricas foram agregadas por AP de residência. O cálculo foi realizado em dias comerciais, às 8h, uma vez que o tempo de deslocamento informado pelo aplicativo considera o trânsito. O tempo e o custo foram calculados tendo como base o uso de transporte público e, para a distância, o deslocamento de carro. O custo foi estratificado de acordo com o valor da passagem em transporte público rodoviário em dólar, com valor de 81 centavos em junho de 2024.

A malha digital dos setores censitários foi obtida do site do Instituto Brasileiro de Geografia e Estatísticas (IBGE)^12^ para a confecção da malha digital dos bairros e das AP.

O projeto foi aprovado pelos Comitês de Ética da Escola Nacional de Saúde Pública Sergio Arouca/Fundação Oswaldo Cruz (CAAE: 94162218.5.0000.5240) e da SMS do Rio de Janeiro (CAAE: 94162218.5.3001.5279).

## RESULTADOS

O município do Rio de Janeiro teve 47 crianças com SCZ, a maioria delas residentes nas zonas norte (AP3) e oeste (AP5); 13,0% (n = 6) evoluíram ao óbito. Entre as 41 crianças que sobreviveram, 83,0% (n = 34) tiveram registros de acompanhamento de puericultura (n = 27, 79,0%) e/ou com especialista (n = 24, 71,0%); e 17,0% (n = 7) não apresentaram registro de atendimento em nenhum dos níveis de atenção à saúde. Apenas 50% (n = 17) tinham o registro de acompanhamento nos dois níveis de atenção à saúde, 29,0% (n = 10) só na APS e 21,0% (n = 7) só na atenção especializada (Figura 1).

**Figura 1:** Atendimento das crianças com síndrome congênita da zika segundo nível de atenção, residentes do município do Rio de Janeiro, 2017.

Fonte: Sistema do Registro de Eventos em Saúde Pública e Sistema de Regulação de Vagas do município do Rio de Janeiro.

APS: atenção primária à saúde; ASS: atenção secundária à saúde.

O número de trajetos percorridos por criança variou entre um e 19 (n = 36 crianças), 67,0% (n = 24) percorreram entre um e quatro trajetos, enquanto 33,0% (n = 11) das crianças tiveram o registro de cinco ou mais trajetos percorridos (Tabela 1). A mediana foi de dois trajetos e a média de quatro trajetos entre a residência e algum serviço de reabilitação no conjunto de todas as crianças. No total foram identificados 143 trajetos percorridos pelas crianças com SCZ, dos quais cada uma teve como destino de uma a três unidades especializadas: 47,0% (n = 17) para três unidades, 28,0% (n = 10) para duas unidades e 25,0% (n = 9) para apenas uma unidade. Quanto aos trajetos: 61,0% (n = 87) para três unidades, 31,0% (n = 45) para duas unidades e 8,0% (n = 11) para uma unidade (Tabela 2).

**Tabela 1:** Número de unidades de destino e trajetos percorridos por crianças com síndrome congênita da zika residentes do município do Rio de Janeiro, 2015 a 2018.

	1 unidade		2 unidades		3 unidades		Crianças	Total
	n	%	n	%	N	%	n	%
Crianças/unidades	9	25	10	28	17	47	36	100
Trajetos								
1 a 4	9		13		15		24	67
5 a 8	1		9		19		5	14
9 a 12	1		22		23		6	17
13 a 19	0		1		18		1	2
Total	11	8	45	31	87	61	36	100

Fonte: Sistema do Registro de Eventos em Saúde Pública e Sistema de Regulação de Vagas do município do Rio de Janeiro.

**Tabela 2:** Número de fluxos percorridos por crianças com síndrome congênita da zika residentes do município do Rio de Janeiro, segundo área de planejamento, 2015 a 2018.

Áreas programáticas	Fluxos								Total
	1		2 a 5		6 a 50		51 a 106		
	n	%	n	%	n	%	n	%	N
1	13	81,25	3	18,75	0	0	0	0	16
2	1	100	0	0	0	0	0	0	1
3	43	69,35	19	30,65	0	0	0	0	62
4	8	80,00	2	20,00	0	0	0	0	10
5	31	53,45	20	34,48	6	10,34	1	1,72	58
Total	96	65,00	44	30,00	6	4,00	1	1	147

Fonte: Sistema do Registro de Eventos em Saúde Pública e Sistema de Regulação de Vagas do município do Rio de Janeiro.

Entre os 143 trajetos, foram registrados 147 fluxos: 65,0% (96) foram percorridos apenas uma vez; 30,0% (44) de duas a cinco vezes; 4,0% (seis) de seis a 50 vezes e 1,0% (um) foi percorrido de 51 a 106 vezes. A distribuição dos fluxos segundo a área de residência foi heterogênea; com predomínio da AP3 e da AP5 (Tabela 2).

Entre as crianças residentes na AP1 foram observados 16 fluxos, dos quais 13 fluxos percorridos apenas uma vez e três fluxos percorridos entre duas e cinco vezes. As unidades especializadas que tiveram o maior número de fluxos estão localizadas em bairros distantes da AP1, na zona sul e na zona norte e apenas um, com dois a cinco fluxos na zona oeste (Tabela 3, Figura 2a).

**Tabela 3:** Tempo gasto, distância percorrida e custo dos trajetos percorridos das crianças com síndrome congênita da zika residentes do município do Rio de Janeiro, segundo área de planejamento, 2015 a 2018.

	Áreas de planejamentos				
	AP1	AP3	AP4	AP5	Total
	**n (%)**	**n (%)**	**n (%)**	**n (%)**	**n (%)**
Tempo					
Até 30 min	0	4 (7)	0	3 (5)	7 (5)
31 a 60 min	5 (42)	17 (30)	2 (25)	4 (7)	28 (21)
61 a 90 min	6 (50)	26 (46)	3 (25)	14 (25)	48 (36)
91 min ou mais	1 (8)	10 (6)	6 (75)	35 (63)	50 (38)
Distância					
Até 10 km	4 (33)	9 (16)	3 (38)	5 (9)	21 (16)
11 a 20 km	5 (42)	26 (46)	0	2 (4)	33 (25)
21 a 30 km	1 (8)	13 (23)	5 (63)	11 (20)	30 (23)
31 a 40 km	1 (8)	9 (16)	0	6 (11)	16 (12)
41 a 50 km	0	0	0	10 (18)	10 (8)
50 km ou mais	1 (8)	0	0	22 (39)	23 (17)
Custo					
1 passagem	2 (17)	12 (21)	2 (25)	3 (5)	19 (14)
2 passagens	0	20 (35)	1 (13)	11 (20)	32 (24)
3 passagens	2 (17)	18 (32)	3 (38)	14 (25)	37 (28)
4 passagens	4 (33)	7 (12)	1 (13)	16 (29)	28 (21)
5 passagens ou mais	4 (33)	0	1 (13)	12 (21)	17 (13)

Fonte: Sistema do Registro de Eventos em Saúde Pública e Sistema de Regulação de Vagas do município do Rio de Janeiro.

**Figura 2:** Mapa de fluxo das crianças com síndrome congênita da zika entre áreas de planejamento de residência e unidades de reabilitação de atendimento no município do Rio de Janeiro, entre 2015 e 2018.

AP: áreas de planejamento; UR: unidades de reabilitação.

Na AP2 houve o registro de apenas um fluxo percorrido uma vez, para um bairro próximo ao local de residência da criança (Figura 2b).

As crianças moradoras da AP3 tiveram 62 diferentes fluxos, 43 percorridos uma única vez e 19 percorridos de duas a cinco vezes. A maioria dos fluxos foi entre bairros que compõem a própria AP, entretanto os fluxos percorridos um maior número de vezes foram em direção à zona sul e oeste da cidade (Tabela 3, Figura 2c).

Entre as residentes da AP4, foram observados 10 fluxos, oito percorridos uma única vez e dois fluxos percorridos de duas a cinco vezes. A maioria dos fluxos ocorreu nos bairros da AP4, porém um percurso com maior número de vezes ocorreu para a zona centro (Tabela 3, Figura 2d).

Entre as crianças residentes da AP5, foi registrada a maior quantidade de fluxos entre as AP do Rio de Janeiro, com 58 diferentes percursos, 31 foram percorridos apenas uma vez, 20 fluxos percorridos de duas a cinco vezes e um único fluxo de 51 a 106 vezes. Uma única criança percorreu 106 vezes a mesma trajetória. A dispersão das trajetórias foi ampla, indo em direção a unidades especializadas nos bairros que compõem a AP e em direção às zonas norte, sul e centro (Tabela 3, Figura 2e).

A mediana de tempo, distância e custo dos percursos entre o endereço de residência das crianças com SCZ foram respectivamente 1h e 18 min, 22,6 km e US$ 2,44 (valor correspondente a até três passagens de ônibus).

Em relação ao tempo, 74,0% (n = 98) dos percursos tiveram mais de 1h de duração (61 min), destes, 50,0% (49) foi entre crianças que residiam na AP5 do Rio de Janeiro, seguido pelos residentes da zona norte com 37,0% (n = 36) dos percursos (Tabela 3).

A maioria (64,0%) dos percursos foi de até 30 km. O mesmo perfil ocorreu entre os residentes das AP1, 3 e 4. Entretanto, as maiores distâncias (mais de 40 km) foram percorridas pelos residentes da AP5 com 24,0% dos percursos (n = 32) (Tabela3).

Quanto ao custo, 62,0% (n = 82) dos percursos custaram às famílias mais de duas passagens. As famílias que tiveram percursos com maior custo foram as residentes da AP5, onde 50,0% (n = 28) dos percursos da região custaram mais de US$ 12 por percurso, representando 21,0% do total de percursos nesta faixa de preço (Tabela 3).

## DISCUSSÃO

Nosso estudo revelou iniquidades no acesso à saúde, especialmente à atenção especializada, agravadas pelas grandes necessidades das crianças com SCZ. Essa dificuldade é mais acentuada para as que residem nas zonas norte e oeste do Rio de Janeiro, regiões mais pobres. Nessas áreas, os deslocamentos em busca de atendimento são mais frequentes, as distâncias entre residências e unidades de saúde são longas e tanto o custo quanto o tempo gasto pelas crianças e suas famílias são maiores, impactando o acesso aos cuidados necessários.

As crianças com idade menor que um ano devem ter garantido o acompanhamento em puericultura até sete dias após o nascimento na APS^9^. Entretanto, 34,0% (14) das crianças com SCZ não tinham o registro de acompanhamento na APS. As crianças com SCZ dependem de procedimentos que variam de menor densidade tecnológica a atendimento especializado^9,13^. No contexto da RAS, compete às unidades dos níveis secundário e terciário o planejamento e a execução de ações de média e alta complexidades, com profissionais especializados e recursos tecnológicos no apoio diagnóstico e terapêutico, não disponíveis na APS^14^.

Um estudo observou insuficiência dos atributos coordenação de cuidado, longitudinalidade, integralidade, orientação familiar na APS para crianças com SCZ^15^. Em nosso estudo, observamos sete crianças sem registro de atendimento, na APS ou nos serviços especializados, indicando inadequação no gerenciamento e acompanhamento pelo gestor local^16,17^.

Apenas 17 crianças foram acompanhadas pela APS e pela rede especializada, conforme preconizado pela Organização Mundial de Saúde (OMS)^9,13^. Há evidências que o acompanhamento e a estimulação precoce reduzem sequelas, aumentam a independência nas funções básicas e melhoram a qualidade de vida^18^. No estado do Rio de Janeiro, 54,3% delas estavam sendo acompanhadas pela APS e 60,3% em algum atendimento especializado^19^. Em duas cidades do nordeste do Brasil, foram constatadas fragilidades para o acompanhamento de gestante pela cobertura insuficiente de pré-natal e falta de cobertura de serviço de saúde com atendimento especializado^20^. Sugere-se que haja gestão do cuidado e processo regionalização inadequados. O processo de regionalização pressupõe o planejamento de ações estratégicas a nível regional, além de reduzir as iniquidades não só quanto à distribuição espacial de unidades de saúde, como também à regulação do acesso mais igualitário de usuários, em conformidade com políticas públicas efetivas^21^.

Com o aparecimento da SCZ, o fluxo de atendimento no município do Rio de Janeiro foi organizado, incluindo os Centros de Reabilitação (CER) e unidades com oferta de diversos serviços de saúde. Entretanto, ainda que o CER tenha ampla oferta de serviços, o estado do Rio de Janeiro não possui CER com todos os tipos de reabilitação^22^. Sendo assim, as crianças que possuem alterações em diversos sistemas, como as com SCZ, precisam se dirigir a várias unidades para assistência integral.

Estudo de análise da distribuição espacial de unidades de reabilitação no estado de Minas Gerais observou desproporção entre as modalidades de reabilitação e distribuição não equânime, refletindo vazios assistenciais^23^. Portanto, é necessária a implementação de estratégias para a redução da mobilidade das crianças com SCZ em busca de atendimento por meio: da avaliação da disponibilidade de unidades de reabilitação, da distribuição das unidades e dos serviços oferecidos, e do aumento de vagas nas regiões de saúde conforme a proporção de pessoas com necessidades especiais no território.

O nosso estudo apontou 143 trajetos diferentes realizados por crianças com SCZ, de acordo com registros da SMS e SISREG, que podem estar subestimados. As crianças realizaram de um a 19 trajetos, entretanto, a maioria percorreu de um a quatro trajetos; isto quer dizer que a maioria percorreu menos trajetos, porém as que tiveram mais trajetos podem ter maior comprometimento clínico. Apenas uma delas completou 19 trajetos, talvez por ser a mais grave e ter realizado mais visitas à unidade de saúde.

A maioria das crianças visitou três unidades de saúde, o que pode estar relacionado à necessidade de serviços e profissionais de diferentes especialidades, nem sempre disponíveis na mesma unidade de saúde, o que também foi observado no estudo de Duttine et al.^5^. Esta situação aumenta os deslocamentos em busca de atendimento, o que pode ser fator desfavorável do acesso ao cuidado.

Embora tenham sido muitos trajetos, 65,0% foram percorridos uma única vez e 30,0% até cinco vezes, mostrando que diversos serviços de saúde fizeram parte da logística de encaminhamento. Nossos resultados correspondem ao registro das redes formais de encaminhamento, entretanto são apontadas redes de apoio informais, o que pode aumentar o acesso, embora possa fragmentar e fragilizar a RAS, o SISREG, a classificação de risco e o acesso igualitário^5^.

Quanto à distribuição dos casos de SCZ, as AP3 e AP5 tiveram o maior número de casos confirmados. São áreas pobres, com distribuição desigual de renda e alta vulnerabilidade social^24^. Estudo que avaliou o risco espacial de desenvolvimento da SCZ observou que a AP3 tem risco relativo elevado espacial, chegando até 7,86^24^. Outros estudos notaram associação entre maior prevalência de SCZ e regiões mais vulneráveis com acesso inadequado ao saneamento básico e disparidades sociais^6,25,26^.

As crianças, residentes em todas as áreas programáticas, apresentaram maior deslocamento na própria área de residência, provavelmente por ser um facilitador de acesso. Embora seja um fator importante para a regionalização do cuidado, uma vez que as pessoas estão percorrendo menores distâncias para serem atendidas, este cenário não foi observado na AP5^27^, zona oeste da cidade, mais distante (em km) do centro, e com limitada disponibilidade de serviços de saúde. Portanto, entre as crianças residentes da AP5 ocorreram mais percursos para fora da área e maior quantidade de fluxos.

Ainda que a maioria dos caminhos percorridos tenha sido dentro da mesma área de moradia, 74% das crianças demoraram mais de 1h entre a residência até o local de tratamento, especialmente os residentes das AP3 e AP5, áreas com maior número de casos e menores índices de desenvolvimento social da cidade^24^.

A maioria dos percursos tinha o custo de duas passagens, totalizando quatro passagens para ida e volta às unidades de saúde. Isto significa que, no preço atualizado para julho de 2024, em média, caso a criança se deslocasse três vezes por semana, o gasto seria de 5,0% do salário-mínimo (US$ 266,62). Valor alto, considerando o perfil socioeconômico das famílias de crianças com SCZ^3,6,28^.

As famílias enfrentam uma rotina exaustiva de cuidados às crianças que apresentam alterações como: dificuldade de deglutição, de sono e repouso, e atraso no desenvolvimento, gerando sobrecarga do cuidador, o que pode ser agravado pela fragmentação do cuidado na rede de saúde^3^. Os cuidados com a criança são prestados, em geral, pelas mães que se dedicam integralmente, sem renda e por vezes abandonadas pelos pais das crianças, em sua maioria, de classe menos privilegiada, pretas e pardas e moradoras de locais precários e com menores índices de desenvolvimento social^24,29^.

Medidas para mitigação do inadequado acesso à RAS e do intenso deslocamento das crianças com SCZ deveriam ser implementadas, tais como: 1) estabelecimento de um fluxo assistencial com integração de todos os pontos da RAS; 2) matriciamento pelas equipes de saúde da família, para um cuidado integral e articulado aos demais níveis de atenção à saúde; e 3) investimento em tecnologias para a realização de interconsultas, teleconsultas, consultas orientadas e telessaúde.

Por se tratar de um estudo com abordagem ecológica, não foi possível avaliar questões individuais como: situação clínica das crianças, nível socioeconômico das famílias e uso de outros serviços de saúde sem o registro pela gestão de saúde local. Outra limitação se refere à estimação de custo de transporte pela ausência de dados individuais. Considerando a complexidade da logística de transporte destas crianças, o gasto das famílias pode ser ainda maior com transporte privado ao invés de público. Embora outras formas de encaminhamento não formais possam ter ocorrido, o SISREG deve organizar a demanda de forma equitativa, transparente e com segurança, com classificação de risco, para o acesso igualitário das crianças à rede de acordo com suas necessidades em saúde^30^, por se tratar da referência formal para regulação. Por fim, considerando a abordagem do estudo, o uso de dados secundários limita o controle sobre a coleta e qualidade das informações.

## CONCLUSÕES

A variação de percursos, a quantidade de fluxos registrados e o número de unidades incluídas nas trajetórias sugerem a complexidade da condição clínica das crianças, desde os cuidados básicos na APS até os procedimentos na atenção especializada. Esta movimentação também sugere uma rede ainda fragmentada, que requer fluxos assistenciais organizados, de forma integrada e compartilhada entre todos os pontos da RAS.

A possível gravidade dos casos de transmissão vertical do OROV, assim como observado nas crianças com SCZ, traz à tona a necessidade de estruturação da RAS para acolher crianças com síndromes congênitas.

Gestores em conjunto com a sociedade devem estabelecer planos nas instâncias de controle social para elaborar estratégias para acolher melhor as demandas das crianças com a SCZ, evitando grandes deslocamentos, subsidiando transporte coletivo, criando unidades de atendimento mais próximas às residências, instituindo o protagonismo da APS com as equipes de saúde da família e equipes multiprofissionais na APS (eMulti)^31^, para estabelecer um cuidado singular e direcionado, com vinculação e coordenação do cuidado compartilhados.

A rede de apoio social também precisa estar imbricada no fluxo de cuidado, garantindo a estas famílias o direito constitucional, seja pelo estabelecimento de uma rede de apoio social, seja por meio de um sistema de transferência de renda efetivo, para contribuir para a melhoria da qualidade de vida.

Diante do indicativo de nossos resultados, estudos observacionais individuais são necessários para investigar estes números, que constituem um retrato do que acontece em outras regiões do país.
